# Development and validation of LAMP primer sets for rapid identification of *Aspergillus fumigatus* carrying the *cyp51A* TR_46_ azole resistance gene

**DOI:** 10.1038/s41598-021-96651-7

**Published:** 2021-08-24

**Authors:** Plinio Trabasso, Tetsuhiro Matsuzawa, Teppei Arai, Daisuke Hagiwara, Yuzuru Mikami, Maria Luiza Moretti, Akira Watanabe

**Affiliations:** 1 School of Medical Sciences, University of Campinas, Campinas, Sao Paulo Brazil; 2 University of Nagasaki, Nagasaki, Japan; 3 Medical Mycology Research Center, Chiba University, Chiba, Japan; 4 Faculty of Life and Environmental Sciences, University of Tsukuba, Tsukuba, Ibaraki Japan; 5 Microbiology Research Center for Sustainability, University of Tsukuba, Ibaraki, Japan; 6 Department of Internal Medicine, School of Medical Sciences, University of Campinas, Rua Tessalia Vieira de Camargo, Campinas, Sao Paulo 126 Brazil

**Keywords:** Fungal genomics, Environmental impact, Fungal infection

## Abstract

Infections due to triazole-resistant *Aspergillus fumigatus* are increasingly reported worldwide and are associated with treatment failure and mortality. The principal class of azole-resistant isolates is characterized by tandem repeats of 34 bp or 46 bp within the promoter region of the *cyp51A* gene. Loop-mediated isothermal amplification (LAMP) is a widely used nucleic acid amplification system that is fast and specific. Here we describe a LAMP assay method to detect the 46 bp tandem repeat insertion in the *cyp51A* gene promoter region based on novel LAMP primer sets*.* It also differentiated strains with TR_46_ tandem repeats from those with TR_34_ tandem repeats. These results showed this TR_46_-LAMP method is specific, rapid, and provides crucial insights to develop novel antifungal therapeutic strategies against severe fungal infections due to *A. fumigatus* with TR_46_ tandem repeats.

## Introduction

Antimicrobial resistance (AMR) was defined by the World Health Organization (WHO) as one of the most critical threats to human health. AMR can compromise our ability to treat infectious diseases, as well as undermining other advances in health care^1^. Although bacterial resistance remains the most common finding in the clinical setting, fungal resistance, especially to azole drugs among filamentous fungi, is relentlessly increasing worldwide^2–4^. Resistance to azole antifungals can be due, in general, to two major genetic mechanisms; point mutation(s) in the *cyp51A* open reading frame with or without tandem repeat (TR) of 34 or 46 base pair (bp) in the promoter region of the gene (TR_34_ or TR_46_) and overexpression of oligonucleotide sequence in the *cyp51A* gene^5^. These mutations and overexpression of the gene confer different levels of resistance^6^. Point mutations result from previous exposure to azole drugs in a clinical setting, such as prophylaxis or therapeutic purposes^7,8^. On the other hand, TR with a point mutation(s) is the aftermath of previous exposure to azole fungicides. In an agricultural setting, azole fungicides are widely used to prevent fungal contamination in a large variety of crop and plant protection, allowing the TR-type resistant strains to emerge in the environments, and the conidia disperse to the air^8,9^. In Brazil, agribusiness represents about 25% of the Brazilian Gross National Product (GNP) (http://www.agricultura.gov.br), and in this scenario, fungicide use has steadily increased over the years. The consumption of pesticides in Brazil grew 190% in 2010, and fungicides corresponded to 14% of this (https://www.ipessp.edu.br).

In the Netherlands, the prevalence of TR-type resistant strains was high. Of 952 clinical *A. fumigatus* strains were collected and included 225 and 98 had TR_34_ and TR_46_, respectively^10^. In another study, TR_34_ L98H and TR_46_ Y121F T289A mutation that occur in patients without previous azole exposure have been reported in Europe, Asia, the Middle East, Africa, and Australia^11^.

Additionally, there are several reports of fatal invasive aspergillosis caused by *A. fumigatus* carrying the TR_46_ in acute myeloid leukemia (AML) patients and hematopoietic stem cell transplant recipients^12^. Higher mortality of patients with invasive aspergillosis caused by azole-resistant strains has been reported^12,13^. Thus, a rapid and specific method to identify the presence of TR would contribute to faster therapeutic decision-making^14^.

As one of the promising diagnostic tools for the azole-resistant *A. fumigatus*, loop-mediated isothermal amplification (LAMP) for the development of improved DNA-based diagnostic kits has been reported^15^. In general, the LAMP method was found to be similar or superior to the standard PCR method, more specific, lower-cost, and easier to perform. LAMP-based approaches have been applied to a wide range of samples, such as whole blood, paraffin-embedded tissues, and various microbial pathogens^16,17^. In this paper, we report a novel LAMP assay method that selectively detects triazole resistant *A. fumigatus* strains due to the presence of double TR46(TR_46_^2^)or triple TR_46_(TR_46_^3^)in the *cyp51A* promoter region.

## Results

### Antifungal susceptibility tests

Drug susceptibilities of 41 *A. fumigatus* strains against azole drugs itraconazole and voriconazole are shown in Table 1. Thirty strains designated as wild type were isolated from clinical specimens, and they were confirmed to be susceptible to itraconazole and voriconazole. The remaining 11 strains (TR_34_ and TR_46_) were resistant to voriconazole, and most of them showed MIC values of > 8 μg/mL against voriconazole. Among the 11 strains, 2 strains (IFM64460 with TR34/L978H and IFM64733 with TR34/LH98H) were resistant to itraconazole, and the remaining 9 strains were susceptible to itraconazole (Table 1).

**Table 1:** *Aspergillus fumigatus* strains used in this experiment and their drug susceptibility profiles against itraconazole and voriconazole.

Strain no	Isolation sources	*cyp51A* genotypes	MIC values (μg/ml)	
			ITCZ	VRCZ
IFM63432^a^	Clinic	TR_46_^2^/Y121F/T289A	4	> 8
BE1-2	Environment (bulb)^b^	TR_46_^2^/Y121F/T289A	2	> 8
BE1-4	Environment (bulb)	TR_46_^2^/Y121F/S363P/I364V/G448S	2	> 8
BE 3-5	environment (bulb	TR_46_^2^/Y121F/T289A	2	> 8
BE 3-6	Environment (bulb)	TR_46_^2^/Y121F/T289A	2	> 8
BE 1-1	Environment (bulb)	TR_46_^3^/Y121F/M172I/T289A/G448S	2	> 8
W1-4	Environment (bulb)	TR_46_^3^/Y121F/M172I/T289A/G448S	2	> 8
W2-12-1	Environment (bulb)	TR_46_^3^/Y121F/M172I/T289A/G448S	2	> 8
IFM64460	Clinic	TR_34_/L98H	> 8	> 8
IFM64733	Environment	TR_34_/L98H	> 8	> 8
3-1-B	Environment (bulb)	TR_34_/L98H/T289A/I364V/G448S	2	> 8
IFM62918^c^	Clinic	Wild	0.5	1
IFM62799	Clinic	Wild	0.5	1
IFM60516	Clinic	Wild	1	1
IFM58402	Clinic	Wild	0.5	0.5
IFM51977	Clinic	Wild	0.25	0.25
IFM60065	Clinic	Wild	1	0.5
IFM61960	Clinic	Wild	0.5	0.5
IFM51748	Clinic	Wild	0.125	0.125
IFM63666	Clinic	Wild	1	2
IFM62520	Clinic	Wild	1	0.5
IFM50999	Clinic	Wild	0.5	0.5
IFM50268	Clinic	Wild	0.25	0.125
IFM55548	Clinic	Wild	0.25	0.25
IFM63311	Clinic	Wild	1	0.5
IFM63355	Clinic	Wild	2	2
IFM60901	Clinic	Wild	0.5	0.5
IFM62674	Clinic	Wild	1	2
IFM62709	Clinic	Wild	0.5	0.5
IFM52659	Clinic	Wild	1	1
IFM57130	Clinic	Wild	0.25	0.125
IFM60814	Clinic	Wild	0.5	0.5
IFM49435	Clinic	Wild	0.25	0.25
IFM61572	Clinic	Wild	0.5	0.5
IFM50669	Clinic	Wild	0.5	0.25
IFM59832	Clinic	Wild	0.5	0.5
IFM55044	Clinic	Wild	0.25	0.25
IFM47670	Clinic	Wild	0.5	0.5
IFM51978	Clinic	Wild	0.5	0.25
IFM58328	Clinic	Wild	0.5	0.5
IFM60369	Clinic	Wild	0.5	0.5

*ITCZ* itraconazole, *VRCZ* voriconazole.

^a^LAMP positive control strain.

^b^obtained from plant bulbs.

^c^LAMP negative control strain.

### Primer design

**Figure 1:**
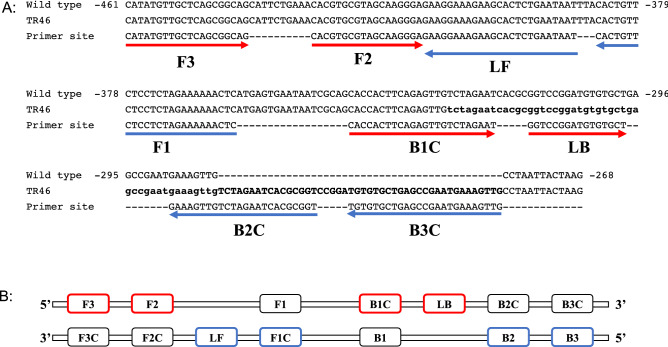
Genetic information for the design of the LAMP primer sets. (**A**) Schematic illustration of *cyp51A* gene showing LAMP primer positions and corresponding sequences of TR46 bp promoter tandem repeat compared to wild-type sequences. (**B**) Primers F3, F2, F1, B1, B2, and B3 show primer sequence positions. Sequences of some primers are complementary, as shown in Table 2. See LAMP primer and methods, which are shown in Refs.^19,20^.

**Figure 2:**
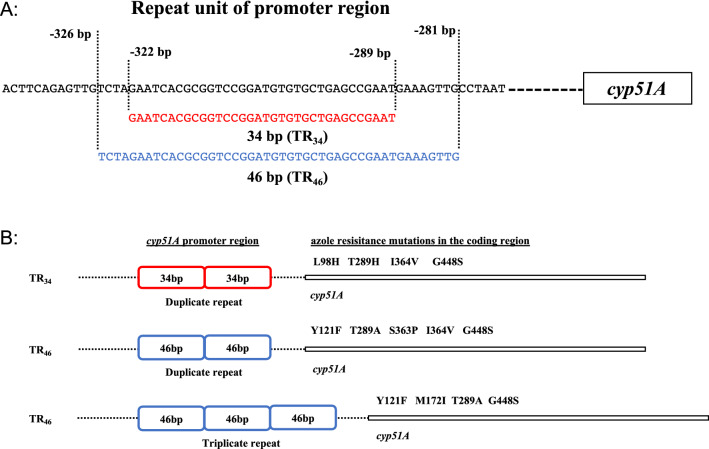
Illustration of tandem repeat regions of *cyp51A* genes used in this experiment. (**A**) Tandem repeat unit of promoter genes of TR_34_ and TR_46_. (**B**) Tandem repeat: 34 bp (double) and 46 bp (double or triple), and *cyp51A* gene associated point mutation place.

**Figure 3:**
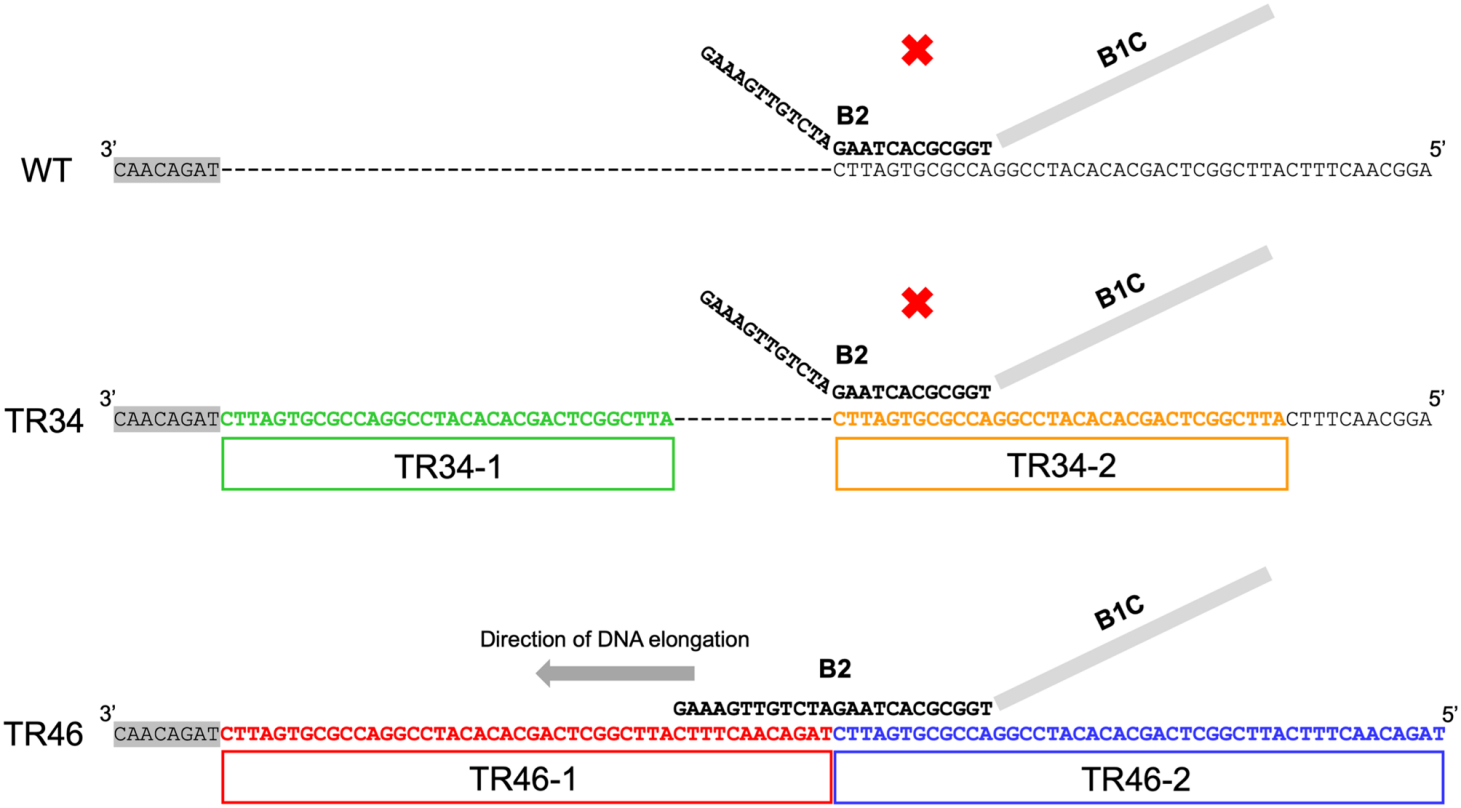
Schematic figure of TR_46_ LAMP primer amplification site in comparison with those of wild type and TR_34_. The nucleotide sequence is targeted for the promoter region for the TR_46_ resistance gene (between TR_46_-1 and TR_46_-2). The sequence in this part corresponds to the B2 sequence in Table 2.

The most crucial step in the LAMP assay is the design of primers. In the LAMP assay, six primers are necessary to amplify the targeted region under isothermal condition. First, we inspected the promoter region (− 461 bp to − 296 bp counted from start codon) of the *cyp51A* gene to select a set of primer sequences that specifically amplify the repeated 46 bp sequence in strains with a TR_46_ mutation (Figs. 1 and 2). To enable specific amplification against repeated TR_46_ sequences, B2 was set on the joint of two 46 bp sequences. Then, another five sequences for primer sets were chosen in the target region, according to the standard criteria, to obtain a specific and rapid LAMP primer set in the LAMP assay. Namely, six primers (F1, F2, F3, B1, B2, B3) that target six specific regions of a DNA template of the TR_46_ gene of *cyp51A* were selected, and in addition, two loop primers (LF, LB) were also chosen to accelerate the reaction (Fig. 1). Several new candidates of LAMP primers were designed based on the above information and their utility tested. From those, one useful LAMP primer set based on the detection of TR_46_ regions in the *cyp51A* gene was selected (Table 2). In this LAMP method, the primers were selected based on the criteria that amplification started within about 30–50 min, and maximum amplification was completed within 70–80 min. Nucleotide sequence of promoter region for resistance gene of LAMP primer sets to detect the resistance gene was shown in Fig. 3. This primer amplifies between consecutive 46 bp sequences between TR_46_-1 bp and TR_46_-2 bp. The base sequence in this part corresponds to the B2 sequence in Table 2.

**Table 2:** Sequence information of newly designed TR46-LAMP primer sets in the present experiment.

LAMP primer names	Sequence (5′ to 3′)
F3	CATATGTTGCTCAGCGGCAG
B3	CAACTTTCATTCGGCTCAGCA
FIP (F1 complementary + F2)	GAGTTTTTTCTAGAGGAGAACAGTG-CACGTGCGTAGCAAGGGA
BIP (B1 + B2 complementary)	CACCACTTCAGAGTTGTCTAGAAT-ACCGCGTGATTCTAGACAACTTTC
LF	ATTATTCAGAGTGCTTCTTTCCTTC
LB	GGTCCGGATGTGTGCTG

### Validation of LAMP primer sets for TR_46_

**Figure 4:**
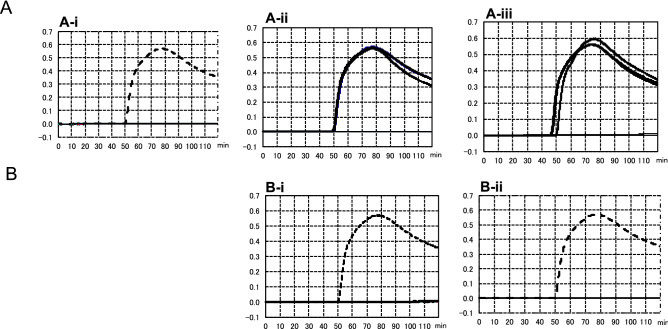
Comparative amplification profiles of *A. fumigatus* wild type and environmental or clinical azole-resistant isolates with or without TR46 double or triple 46 bp promoter repeats in *cyp51A* gene by a newly developed LAMP primer sets. The dotted curve shows the amplification by control strain (IFM 63432). (**A**-**i**) DNA amplification profiles using 30 strains of *A. fumigatus* wild type. DNA amplification was not confirmed in all wild-type strains tested (30 strains). Among 30 wild-type strains, IFM 62918 strain was used as a negative control strain (no amplification). (**A-ii**) DNA amplification was confirmed by five TR_46_^2^ strains (IFM63432, BE1-2, BE1-4, BE3-5, BE3-6), which have double 46 bp promoter repeats. (**A-iii**) DNA amplification was confirmed by three TR_46_^3^ strains (BE1-1, W1-4, W2-12-1), which have triple 46 bp promoter repeats. (**B-i**) DNA amplification was not confirmed by two TR_34_^2^ strains (IFM64460, IFM64733), which have duplicate 34 bp promoter repeats with one mutation in the one coding region (L98H). The dotted line shows amplification by the control strain. (**B-ii**) DNA amplification was not confirmed by one TR_34_^2^ strain (3-1-B), which has duplicate 34 bp promoter repeats with multi-mutations in the four coding regions (L98H/T289A/I364V/G448S). The dotted line shows amplification by the control strain.

**Figure 5:**
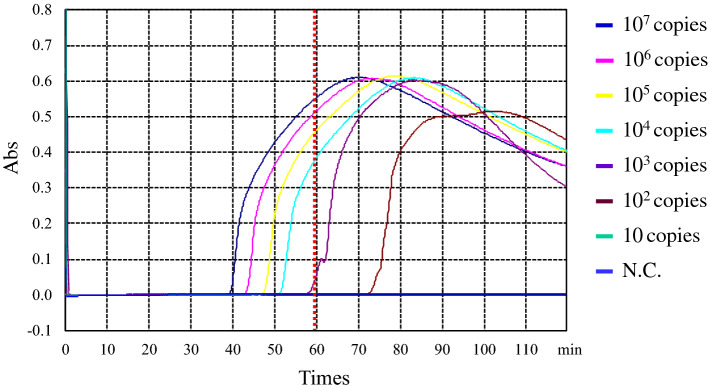
Experimental results of the detection limit of the TR_46_ LAMP assay. The detection limit reaction was carried out using 10^7^ to 10 copies of plasmid DNA per reaction. The detection limit was measured within 60 min.

The specificity of the primer sets was tested using various types of *A. fumigatus* strains, such as wild isolates and environmental or clinical azole resistance isolates (Fig. 4). In this study, IFM63432 and IFM62918 strains were used as positive and negative control strains, respectively. As shown in Fig. 4A-i, TR46 LAMP primer could not amplify the DNA from 30 strains of azole drug-susceptible clinical isolates of *A. fumigatus*. However, the start of the LAMP amplification in the positive control strain of *A. fumigatus* strains (IFM 63432) was at around 50 min. On the other hand, TR_46_ LAMP primer could amplify DNA from *A. fumigatus* strains carrying the duplicate 46 bp promoter repeat in *cyp51A* gene (IFM63432, BE1-2, BE1-4, BE3-5, BE3-6) as shown in Fig. 4A-ii. This result suggests that the present LAMP primer could amplify the four TR_46_^2^ strains harboring the TR46 resistant mutation (TR46/Y121F/T289A). It was also confirmed that TR_46_ LAMP primer could amplify DNA of *A. fumigatus* strains carrying three tandem repeats (TR_46_^3^) (BE1-1, W1-4, W2-12-1) (Fig. 4A-iii). When this TR_46_ LAMP primer was tested for three TR_34_^2^ strains (Table 1), namely strains IFM64460 and IFM64733 (with mutation of TR_34_/L98H) and strain 3-1-B (with mutations of TR_34_/L98H/Y289/T289A/I364V/G448S), DNA amplification was not observed (Fig. 4B-i,B-ii). These results also suggested that the present LAMP primer could not detect TR_34_^2^ drug-resistant strains regardless of their point mutation site in the *cyp51A* gene (Fig. 4B-i,B-ii). These studies confirmed that the newly established TR_46_ LAMP primer set was specific for *A. fumigatus* strains with TR of double or triple 46-bp promoter tandem repeats in the *cyp51A* gene. The sensitivity of the TR_46_ LAMP assay was verified. The detection limit was 1 × 10^4^ copies per reaction in 60 min. In the 80 min reaction, 10^2^ copies per reaction were also detected (Fig. 5).

## Discussion

Azole antifungals mainly inhibit the ergosterol biosynthetic pathway by targeting the cytochrome P450-dependent enzyme lanosterol 14-α-demethylase, encoded by *cyp51A* in molds. Resistance to this class of drugs in the major human pathogen *A. fumigatus* is emerging and reaching levels to prevent their clinical use^6^. Advances in recent molecular genetic technologies such as real-time PCR have introduced various proper diagnostic assay methods into the fields in azole-resistant mechanism analysis. The LAMP assay described here has advantages of high sensitivity and specificity, low costs, and short amplification time. In addition, there have been no reports using LAMP techniques to study azole-resistant mechanisms in *A. fumigatus* by the strains with TR_46_ in the *cyp51A* promoter region.

Recently Yu Shan-Ling et al.^18^ reported a similar rapid technique to detect azole-resistant strains due to amplification of a TR of a 34 bp (TR_34_) and a 46 bp (TR_46_) within the promoter region of *cyp51A* of *A. fumigatus.* However, here we used a newly designed TR_46_ LAMP primer set different from those reported by Yu Shan-Ling et al.^18^. Compared to experiments such as Yu Shan-Ling et al.^18^, our method used adjusted genomic DNA (2 ng/μL) and can detect TR_46_ strains when resistance is confirmed, and this leads to a simple identification method of *A. fumigatus* carrying the TR_46_ in the *cyp51A* promoter region, in routine clinical practice.

There is a difference in MIC values between strains with TR_46_ and strains with TR_34_^19^. Therefore, the importance of detecting TR_46_ lies in the fact that strains of *A. fumigatus* harboring TR_46_ are resistant to voriconazole but not to itraconazole. Two TR_34_ strains (IFM64460: TR_34_/L98H and IFM64733: TR_34_/L98H) are highly resistant to voriconazole^19^ but not to itraconazole. Further detailed drug susceptibility mechanism study against TR_34_ strain (3-1-B: TR_34_/L98H/T289A/I364V/G448S) is of interest.

The high specificity and rapidity of the LAMP assay are achieved by applying four primers that target six regions of a DNA template, and two loop primers (LF, LB) to accelerate the reaction. In this study, we succeeded in designing valuable TR_46_ LAMP primer sets to detect specifically a TR_46_ within the promoter regions of azole-resistant *A. fumigatus*. Furthermore, the designed primer sets could differentiate azole-resistant TR_46_ strains from the TR_34_ strains and wild type strains. There was no cross reaction of the assay with neither the TR_34_ type nor the wild type. Further studies on the preparation of specific primers that can distinguish between TR_34_ strains and wild type strains regardless of the results of drug susceptibility testing are needed. To our knowledge, this is a new and helpful report of a detection method for one of the most prevalent *cyp51A* resistant gene TR_46_ in *A. fumigatus* azole-resistant strains.

Recently, the strain consisting of the four repeats of 46 bp of the promoter region was reported in the Netherlands (TR_46_^4^)^20^. The LAMP primer we designed was able to detect both two copies of the TR46 tandem repeat and three copies of the TR_46_. Moreover, these amplification curves (as well as the starting point) were similar. The BIP (B1 + B2 complementary; Fig. 1) of the primer we designed is TR-specific. B1 is designed at the boundary where the repeat unit is inserted, and B2 is designed at the boundary between the repeat units. In addition, B3 is designed on a repeat unit. Based on the results of strains having double repeat and triple repeat, it was suggested that the primer used this time may be able to detect even if the number of repeats increases, such as TR_46_^4^.

It is widely known that exposure to azole fungicides resulted in the emergence of azole-resistant strains with tandem repeats in the promoter region of *cyp51A* gene^8,9^. For this reason, epidemiological studies such as the incidence of azole-resistant strains in the environment are essential. Many environmental and clinical isolates need to be screened to generate epidemiological data, such as the frequency of detection of azole-resistant *A. fumigatus*. The method developed in this study would be an easy-to-use screening procedure.

Since the LAMP assay developed in the present study is a one-step and rapid detection method, coupled with its high reliability and ease of use, it can prompt detect specific drug-resistant genes due to TR_46_ in *A. fumigatus* in the clinical laboratory setting. Thus, early detection of infections due to TR_46_ drug-resistant strains in *A. fumigatus* might be helpful to guide the early start of corrective and effective antifungal therapy.

## Methods

### Aspergillus isolates and MIC determination by broth microdilution test

Forty-one strains, including thirty-three from the clinical setting and eight environmental (plant bulbs) isolates^21^ of *A. fumigatus,* were provided through the National Bio-Resource Project (NBRP), Japan (http://www.nbrp.jp/); source and drug susceptibility are shown in Table 1.

### DNA preparation and extraction

Fungal strains were cultured on Sabouraud dextrose agar. Genomic DNA was extracted from overnight cultures of *A. fumigatus* mycelia by the urea-phenol method. Mycelia were mixed with 0.5 mm size glass beads, 0.5 ml of PCI (phenol/chloroform/Isoamyl alcohol) solution and 0.5 ml DNA extraction buffer (50 mM Tris–HCl, pH 8.0, 20 mM EDTA, 0.3 M NaCl, 0.5% SDS, 5 M urea), and disrupted by Fast Prep FP100A (MP-Biomedicals, Santa Ana, USA) for 3 cycles of 30 s each at a speed of 4.0 m/s. After centrifugation, the upper layer was transferred to a new tube and subjected to ethanol precipitation. The resulting DNA pellet was suspended in 100 μL TE buffer. DNA concentration was determined by the methods described in our previous paper^22^.

All *A. fumigatus* strains were submitted to antifungal susceptibility tests according to the CLSI M38 protocol (https://clsi.org/standards/products/microbiology/documents/m38/), using Eiken Dried Plates (9DEF47, Eiken Chemical Co., Tokyo, Japan).

### LAMP-method

LAMP was performed as described in our previous studies^23^. TR_46_ LAMP primers were designed based on the target promoter region sequences of the *cyp51A* gene of *A. fumigatus*, which includes tandem repeats in the promoter region containing TR_46_ mutant alleles. The sequence of the *cyp51A* gene was downloaded from NCBI Gen-Bank (https://www.ncbi.nlm.nih.gov/genebank, accession numbers AF222068 for wild type, MH231595.1 for TR_34_, and MH040305.1 for TR_46_). In total, a 184-bp nucleotide alignment (Fig. 1) was used for TR_46_ LAMP primer design by the protocol of the Eiken Company (Primer Explorer V5, Eiken Chemical Co. Ltd, Tokyo. Japan). LAMP primers are composed of six primers recognizing eight distinct regions. LAMP reactions were performed with a Loopamp DNA amplification kit using reaction mixtures composed of 40 pmol each of primers FIP and BIP, 5 pmol each of primers F3 and B3, 20 pmol each of primers LF and LB, 12.5 mL × 2 reaction mixture, 1 μl *Bst* DNA polymerase, 2 μL DNA sample and distilled water up to a final volume of 25 μL (Eiken Chemical Co., Ltd., Tokyo, Japan). The LAMP reactions were analyzed by a real-time turbidimeter (Loopamp EXIA; Eiken Chemical Co.) and were conducted at 63 °C, for 120 min and then heated at 80 °C for 2 min to terminate the reaction. The start of amplification of LAMP products at 30 to 50 min in the graph suggested the positive reaction due to the presence of corresponding 46 bp tandem repeats of *cyp51A* gene. Since overall reaction can be obtained within 2 h, prompt drug therapy can be deployed within a short time. To check the detection limit of TR_46_ specific LAMP primers, the plasmid DNA was used. To construct plasmid contained TR_46_ and *cyp51A* gene sequences, we cloned the alleles using the shuttle vector pCB1004. Genomic DNA of IFM63432 was used as template to clone the alleles. The cyp51 coding region including approximately 1 kb fragments upstream and downstream were amplified by PCR using the primers pCB1004_Hind_cyp51A-F (5’-aggaattcgatatcaTAGAATGAGTGAGCTGATTT-3’) and pCB1004_Kpn_cyp51A-R (5’-gggcgaattgggtacCAGGTTTTCGCACGAGCTTCTCC-3’). Amplified DNA fragments were fused into pCB1004 digested with *Hin*dIII and *Kpn*I, by In-Fusion H Cloning Kit (Takara Bio, Otsu, Japan). The size of plasmid DNA was 8164 bp.
